# Analysis of histone modifications in key cellular subpopulations in the context of azoospermia using spermatogenic single-cell RNA-seq data

**DOI:** 10.3389/fbinf.2025.1626153

**Published:** 2025-07-18

**Authors:** Qiu Wang, Hong Yang, Fang Li, Song Ge, Ling Ji, Xiaofeng Li

**Affiliations:** ^1^ Department of Laboratory Medicine, Peking University Shenzhen Hospital, Shenzhen, China; ^2^ Guangdong Provincial Clinical Research Center for Laboratory Medicine, Guangzhou, China

**Keywords:** male infertility, histone modification gene, non-obstructive azoospermia, single-cell RNA sequencing, Leydig cells

## Abstract

**Introduction:**

The molecular underpinnings of non-obstructive azoospermia (NOA), a severe form of male infertility characterized by the absence of sperm in the ejaculate, remain unclear.

**Methods:**

In this study, we demonstrate the role of histone modifications within specific testicular cell subpopulations in NOA using single-cell RNA sequencing (scRNA-seq) data.

**Results:**

Based on scRNA-seq analysis of the data acquired from the Gene Expression Omnibus (GSE149512), we identified nine distinct cell types and revealed significant compositional differences between the NOA and control testicular tissues. In contrast to the high prevalence of spermatogenic cells in the controls, endothelial, testicular interstitial, and vascular smooth muscle cells, as well as macrophages, were enriched in NOA. Furthermore, our analyses revealed considerable enrichment of histone modificationrelated genes in Leydig cells, peritubular myoid (PTM) cells, and macrophages in the NOA group. HDAC2, a pivotal regulator of histone acetylation, exhibited significant upregulation. Functional pathway analysis implicated these genes in critical biological processes, including nuclear transport, RNA splicing, and autophagy. We quantified the activity of histone modificationrelated genes using AUCell and identified distinct Leydig cell subpopulations characterized by unique marker genes and functional pathways, underscoring their dual roles in histone modification and spermatogenesis. Additionally, cellular communication analysis via CellChat demonstrated altered interaction dynamics across cell types in NOA, particularly in Leydig and PTM cells, which exhibited enhanced interactions alongside differential activation of the WNT and NOTCH signaling pathways.

**Discussion:**

These findings suggest that aberrant histone modifications in specific cellular subpopulations may drive disease progression, highlighting potential targets for diagnostic and therapeutic strategies. This study offers novel insights into the molecular mechanisms of NOA and provides a basis for future research on advanced male reproductive health.

## Introduction

Infertility affects approximately 15% of couples of reproductive age worldwide (Carson and Kallen, 2021), with 30% of cases associated with male factors (Saha et al., 2021). Various factors have been attributed to male factor infertility, but 30% of men exhibit reduced sperm quality without any identifiable reason, thereby delineating the condition of idiopathic male infertility (Boeri et al., 2024). It is categorized into the following subtypes: idiopathic oligospermia (sperm concentration <20 million per milliliter), idiopathic asthenozoospermia (sperm concentration >20 million but low motility), idiopathic teratozoospermia (normal sperm concentration and motility, significant percentage of abnormal sperm in the ejaculate), and idiopathic non-obstructive azoospermia (NOA; no sperm in the ejaculate).

Sperm production is a complex process that involves numerous genes. Over 800 genetic loci have been associated with male reproduction in different mammalian species (Wagner et al., 2023). Several genetic factors have been implicated in infertility, including abnormalities in mitochondrial DNA (mtDNA) (Sang et al., 2023). Multiomics, combined with systems biology, facilitates the elucidation of potential disease mechanisms and development of new diagnostic and treatment strategies. Several studies have revealed that epigenetic mechanisms such as DNA methylation, residual histone modifications, chromatin remodeling, and non-coding RNA-mediated regulation potentially impact male infertility and the outcomes of assisted reproductive technology (Shacfe et al., 2023; Marquardt et al., 2023; Tahmasbpour Marzouni et al., 2022; Hong et al., 2022a; Hong et al., 2022b). Healthy sperm production requires a series of histone modifications associated with the transformation of transition proteins and testis-specific histone variants into sperm proteins. Altered post-transcriptional modifications (PTMs) of histones have been reported in men with abnormal semen parameters. These changes in PTMs include altered H4 acetylation, as well as H4K20 and H3K9 methylation, compared to those detected in normal sperm samples (van Nuland and Gozani, 2016). Sirtuins are NAD + -dependent deacetylases that catalyze the removal of acetyl groups from lysine residues on protein substrates through a nicotinamide adenine dinucleotide (NAD^+^)-consuming reaction (Sauve and Youn, 2012). Zmynd15 encodes a MYND-domain zinc-binding transcriptional repressor involved in spermatogenesis, functioning through histone deacetylase. Its knockout or mutation can lead to male infertility in mice (Wen et al., 2023; Yan et al., 2010). Germ cell-specific Sirt1 deletion in male mice disrupts chromatin condensation during gametogenesis due to reduced H4 hyperacetylation (K5, K8, K12), leading to reduced fertility (Gunes et al., 2025). Thus, Histone modifications are crucially involved in sperm production; any imbalance in this process may contribute to or primarily cause infertility.

Patients with NOA exhibit abnormalities in spermatogenesis. While normal sperm production or quality is a good predictor of health outcomes in these patients. Patients with NOA more frequently exhibit infertility-related symptoms compared to those without this condition. However, effective drug therapies to improve normal sperm production in patients with NOA are unavailable. In this study, we aim to identify subpopulations of cells with histone modification activity in patients with NOA and their association with the infertility-related symptoms based on single-cell data acquired from both healthy controls and patients with NOA. This approach can potentially allow for a deeper understanding of the pathobiology of NOA.

## Materials and methods

### Dataset acquisition and preprocessing

We obtained scRNA-seq data from the Gene Expression Omnibus (GEO) database (GSE149512), which contained 96,524 cells, including 10 normal (Control) and 7 azoospermia samples. The R package Seurat was used for further analysis of single-cell data. Cells were filtered based on the following threshold parameters: the total number of expressed genes was between 500 and 9,000, and the total count of Unique Molecular Identifiers (UMIs) was between 0 and 35,000. The IntegrateData function within the Seurat package was used for batch correction. After quality control, we obtained 87,982 high-quality cells. After normalizing the data, all highly variable genes were identified. Subsequently, principal component analysis with highly variable genes was used to identify significant principal components (PCs). Twenty PCs were selected for dimensionality reduction and clustering. The cell type was determined in each cluster based on marker genes for each cell type, following a previous report (Zhao et al., 2020). A total of 431 histone modification-related genes were extracted from the Gene Set Enrichment Analysis website (https://www.gsea-msigdb.org/gsea/index.jsp; accessed prior to 1 Sep 2020) (Supplementary Table S1) (Chen et al., 2021).

### Activity score of cell DEGs

We calculated the differentially expressed genes (DEGs) between different cell types in GSE149512 using the FindAllMarkers function in the R package Seurat. Next, the activity score for each cell was estimated using the R package AUCell, based on a set of 110 DEGs obtained from the intersection of DEGs for each cell type and histone modification-related genes. This set of 110 DEGs was used as the input data to calculate the area under the curve (AUC) values. According to the AUC values, a gene expression ranking was established for each cell. The AUC reveals the proportion of highly expressed genes within a gene set per cell. Cells comprising several genes from the gene expression set exhibited a higher AUC value than those presenting fewer genes. The active-cell population was determined by calculating the optimal threshold ($aucThr) for the current gene set using the function AUCell_exploreThresholds.

### Functional enrichment analysis

We used the R package clusterProfiler to perform Gene Ontology (GO) and Kyoto Encyclopedia of Genes and Genomes (KEGG) functional enrichment analyses of DEGs in each cell subpopulation, filtering significantly enriched pathways and functions based on a threshold of P < 0.05.

### Screening DEGs

The FindAllMarkers function in the R package Seurat (with test. use = “wilcox”) was used to identify specific marker genes for each cell cluster based on normalized UMI counts. For genes differentially expressed between cell subpopulations, only those detected in at least 10% of the cells were considered, with thresholds of adjusted p-value (adj.p) <0.05, and absolute log2 fold change (|log2FC|) > 0.25.

### Construction of the protein-protein interaction network

We constructed a protein-protein interaction (PPI) network using the STRING database to identify markers closely related to spermatogenesis.

### Cellular communication

We integrated the gene expression data of the azoospermia and control groups using the R package CellChat and compared the presumed cell-cell communication modules between cell types and interactions between significantly altered cell types in the azoospermia and control groups.

### Immunofluorescent staining

Human testicular biopsy tissue samples were obtained from Peking University Shenzhen Hospital, where routine testicular biopsies were performed. Samples were fixed in 4% paraformaldehyde (PFA) overnight, and then embedded in paraffin. Paraffin-embedded samples were sectioned into 3 μm-thick slices. Subsequently, the sections were dewaxed and dehydrated using standard protocols. Antigen retrieval was performed by heating the sections in a microwave oven with 0.01 M sodium citrate buffer (pH 6.0). To block non-specific antibody binding, sections were incubated with 5% bovine serum albumin for at least 30 min at room temperature. Samples were incubated overnight at 4°C with the following primary antibodies diluted in phosphate-buffered saline: rabbit anti-EZH2 (Proteintech, 21,800-1- AP, 1:500), mouse anti-IL-6 (Proteintech, 66146-1-Ig, 1:500), and rabbit anti-HDAC2 antibody (2540S,1:500). The sections were washed and incubated with secondary antibodies for 2 h at room temperature. The secondary antibodies used were Alexa Fluor 488 (Invitrogen)-conjugated donkey anti-rabbit immunoglobulin (Ig) G and Alexa Fluor 594 (Invitrogen)-conjugated donkey anti-mouse IgG, followed by PBS three washes. Subsequently, the samples were counterstained with 4′,6-diamidino-2-phenylindole (DAPI), mounted using Vectashield mounting medium (Vector Laboratories, H-1200), and visualized under confocal microscopy (Leica, STELLARIS 5).

### Reverse transcription and real-time polymerase chain reaction (PCR)

Total RNA was extracted using an FFPE RNA Kit (OMEGA, R6954) following the manufacturer’s instructions. RNA concentration was measured using a Nanodrop Spectrophotometer (Thermo); 1–4 μg RNA was reverse transcribed using High-Capacity cDNA Reverse Transcription kits (Applied Biosystems). Real-time PCR was carried out on an Applied Biosystems Q5 96-well Real-Time PCR System using SYBR Green master mix (Takara). The following primers were used: *HDAC2*, F- CTCATGCACCTGGTGTCCAGAT, R- GCTATCCGCTTGTCTGATGCTC; *GAPDH*, F- GTCTCCTCTGACTTCAACAGCG, R- ACCACCCTGTTGCTGTAGCCAA. Gene expression was analyzed using the 2^−△△CT^ method (Livak and Schmittgen, 2001).

## Results

### The study design workflow is depicted in Figure 1

#### Single-cell RNA sequencing data preprocessing

We obtained a NOA single-cell dataset from the GEO database (GSE149512), which included 17 samples; the control and azoospermia groups included 10 and 7 samples, respectively. After quality control, we obtained 87,982 high-quality cells. The clustering analysis and annotation of the single-cell data identified 9 cell types, including 2,310 endothelial cells, 25,374 testicular interstitial cells, 5,166 macrophages, 11,201 peritubular myoid cells, 13,986 Sertoli cells, 13,550 spermatogenic cells, 5,353 spermatogonia, 8,334 spermatids, and 2,708 vascular smooth muscle cells (Figure 2A; Supplementary Figure S1). Moreover, comparative analysis of the cell infiltration in diseased and control tissues revealed a significantly higher infiltration level of endothelial cells, testicular interstitial cells, macrophages, and vascular smooth muscle cells in the NOA group compared to controls. Conversely, control tissues exhibited a significantly higher infiltration level of spermatogenic cells, spermatogonia, and spermatids than their counterparts in the azoospermia group (Figures 2B,D). Additionally, we presented the expression levels of marker genes in each cell cluster (Figure 2C). SCARA5, CST9L, CCDC168, KRT17, LUZP4, CARTPT, C1QC, RERGL, and SELE were primarily expressed in testicular interstitial cells, Sertoli cells, spermatogenic cells, peritubular myoid cells, spermatogonia, spermatids, macrophages, vascular smooth muscle cells, and endothelial cells, respectively (Figure 2C) .

**FIGURE 1:**
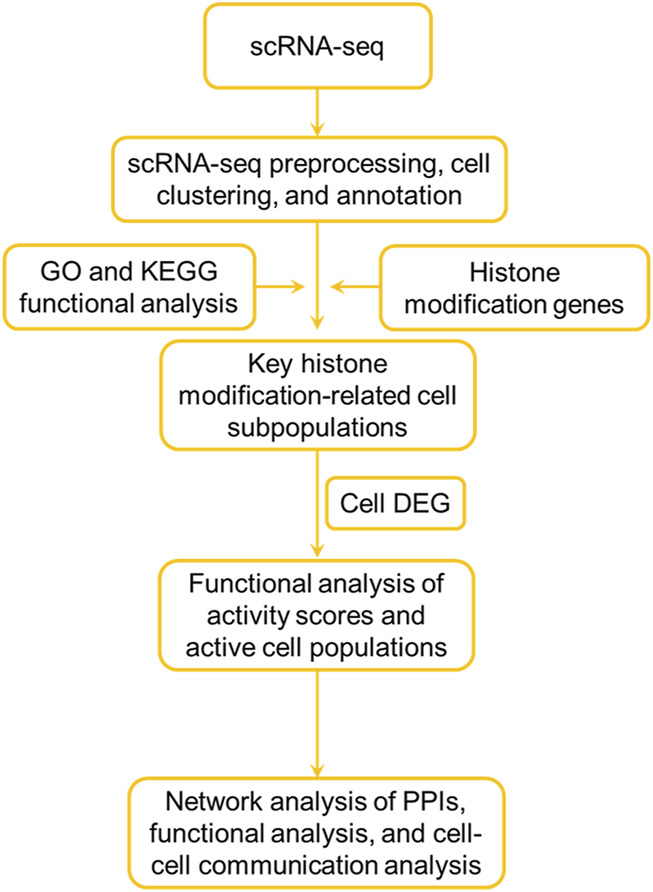
Flowchart of the analysis.

**FIGURE 2:**
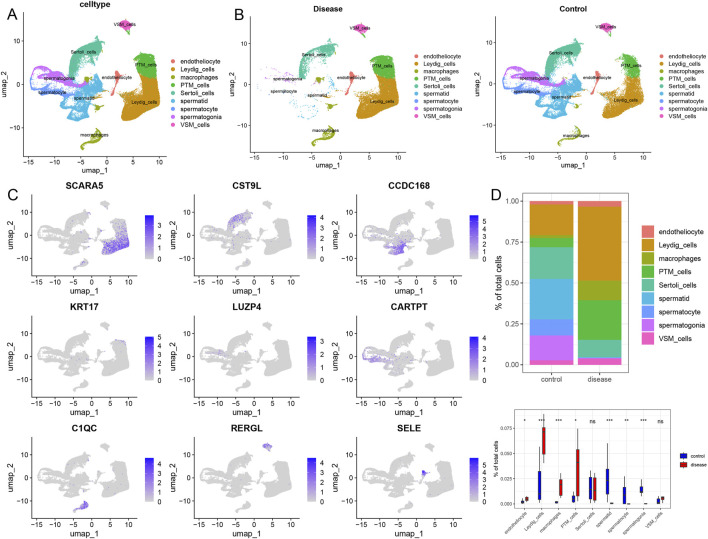
Landscape of the single-cell transcriptome in GSE149512. **(A)** UMAP diagram of cellular subtypes. **(B)** Distribution of cellular subtypes in non-obstructive azoospermia and control samples. **(C)** UMAP diagram reflecting the expression of marker genes in nine different cell types. **(D)** Comparative proportions of each cell type in the non-obstructive azoospermia and control groups. Statistical analysis was performed using the Wilcoxon rank-sum test. Endotheliocyte: Endothelial cell, Leydig cells: Leydig cells of the testis, Macrophages: Macrophages, PTM cells: Peritubular myoid cells, Sertoli cells: Sertoli cells, Spermatid: Spermatid, Spermatocyte: Spermatocyte, Spermatogonia: Spermatogonia, VSM cells: Vascular smooth muscle cells.

#### The heterogeneity of histone modifications in infertility and identification of key cellular subpopulations

**FIGURE 3:**
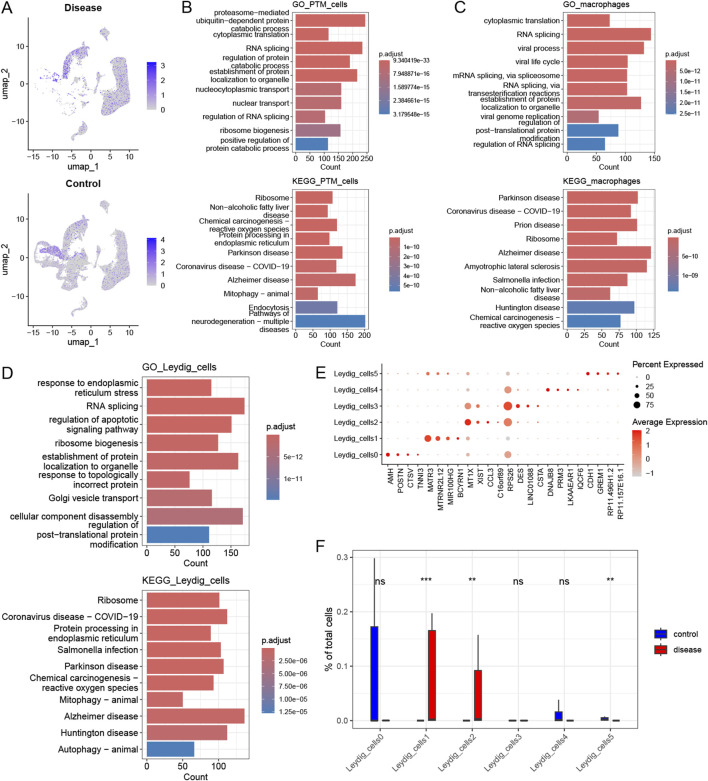
Heterogeneity of histone modifications and identification of key cellular subpopulations. **(A)** Expression profile of the histone modification gene HDAC2 across different cell types in the disease and control groups. **(B)** Functional pathway enrichment analysis for PTM_cells. **(C)** Functional pathway enrichment analysis for the macrophages. **(D)** Functional pathway enrichment analysis for the Leydig_cells. **(E)** Differential expression of selected marker genes within each Leydig_cell subpopulation. **(F)** Variation in the proportion of histone modification-related cellular subpopulations between the control and disease groups.

Based on a previous report, 431 genes related to histone modifications were included in this study (Chen et al., 2021). Subsequently, the expression of histone modification genes in different cell types in the disease and control groups was explored; PTM cells, macrophages, and Leydig cells were significantly enriched in the disease group. Among them, 136, 51, and 110 upregulated genes related to histone modifications were detected in PTM cells, macrophages, and Leydig cells (with a differential gene screening threshold of p < 0.05 and log2FC > 0.25), respectively. Among these genes, *HDAC2*, which is involved in histone modification, was significantly upregulated in the Leydig and PTM cells of the disease group (Figure 3A). Next, functional pathway enrichment analysis of the DEGs in PTM cells, macrophages, and Leydig cells from the control and disease groups revealed that the DEGs in PTM cells were enriched in functions such as nuclear transport and RNA splicing (Figure 3B). The differential genes in macrophages were enriched in post-translational protein modification and RNA splicing (Figure 3C), whereas those in Leydig cells were enriched in functions and pathways such as autophagy-animal and regulation of apoptotic signaling pathway (Figure 3D), which are closely related to clinical phenotypes. Therefore, we consider Leydig cells to be key cellular subpopulations related to histone modifications. We reclassified Leydig cells, identified six cellular subpopulations, and identified the marker genes for each subpopulation (Figure 3E). Subsequently, we explored the differences in the proportions of each histone modification-related cellular subpopulation between the control and disease groups, and found that the cellular subpopulations Leydig_cells1, Leydig_cells2, and Leydig_cells5 significantly differed between the disease group and the control group (Figure 3F).

#### Histone modifications are closely associated with the activation of crucial cell subpopulations and spermatogenesis

**FIGURE 4:**
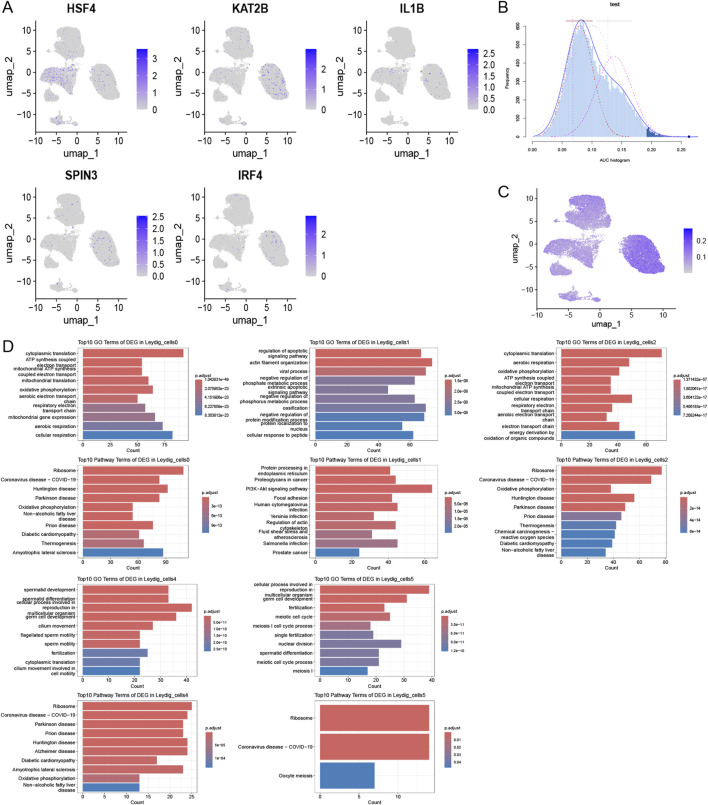
Activation of histone modification in key cellular subpopulations. **(A)** Comparative histone modification-related gene expression patterns across different cell types. **(B)** Activity scoring of the 110-DEG gene set with a selection threshold of 0.1267. **(C)** UMAP plot based on the activity scores of the DEG set for each cell. **(D)** Histone modification-related functions of key cellular subpopulations within the active-cell subpopulation.

**FIGURE 5:**
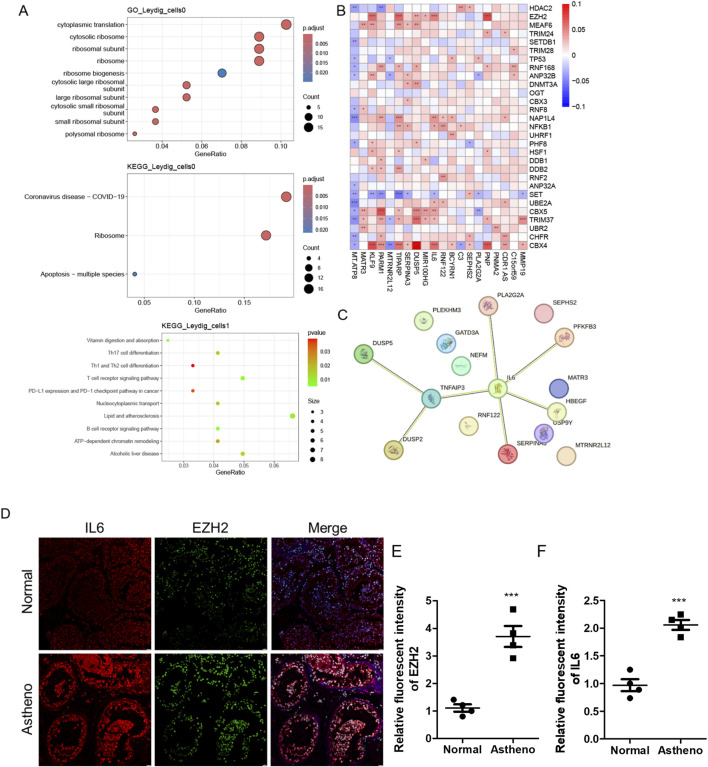
Histone modifications are intricately associated with the activation of crucial cell subpopulations and spermatogenesis. **(A)** Functional and pathway enrichment analysis of the marker genes of the active-cell population in the disease and control groups. **(B)** Protein-protein interaction network reflecting the interactions among the marker genes of the cellular subpopulations closely related to spermatogenesis in the disease group. **(C)** Expression of markers for cellular subpopulations is closely related to spermatogenesis and histone modifications. **(D)** IF staining analysis reveals the expression of IL6 or EZH2 in normal and pathological testicular tissues. **(E,F)** ImageJ-based analysis of the relative fluorescence intensity of **(E)** and IL6 (F; n = 4). Data are presented as the mean value ±SEM. Statistical significance between groups was determined using a two-tailed unpaired t-test. ***, p < 0.001.

We identified DEGs by intersecting the histone modification-related genes in key cellular subpopulations (Leydig_cells) between the disease and control groups, considering a differential expression threshold of p < 0.05, and log2FC > 0.25. We obtained 110 DEGs and explored the expression levels of a few selected DEGs (Figure 4A). We calculated the activity score for each cell using the R package AUCell and scored the gene sets related to histone modifications. Cells expressing multiple genes in the gene set exhibited higher AUC values than those expressing fewer genes. When the AUC value threshold was set to 0.1267, 6390 Leydig_cells, including 569 Leydig_cells0 and 5,671 Leydig_cells1, showed relatively high AUC values (Figure 4B) and were considered active cells (Figure 4C). Based on the active-cell population, we identified the DEGs for each histone modification-related key cellular subpopulation and determined the cell population functions (Figure 4D). The DEGs in Leydig_cells0 were primarily enriched in functions and pathways, such as oxidative phosphorylation and mitochondrial translation. In Leydig_cells1, DEGs were mainly enriched in functions and pathways, such as the PI3K-Akt signaling pathway, actin filament organization, and regulation of the apoptotic signaling pathway. The DEGs in Leydig_cells2 were mainly enriched in functions and pathways, such as oxidation of organic compounds and oxidative phosphorylation. The DEGs in Leydig_cells4 were mainly enriched in functions and pathways such as spermatid development and differentiation, whereas those in Leydig_cells5 were mainly enriched in functions and pathways such as spermatid differentiation. Subsequently, we calculated the marker genes for each subpopulation of active cells in the disease and control, and performed GO and KEGG functional enrichment analysis and pathway enrichment analysis of the differential cellular subpopulation gene sets in the disease (Figure 5A). Leydig_cells0 were mainly enriched in functions and pathways, such as ribosomes and cytosolic ribosomes. Leydig_cells1 was primarily enriched in immune-related pathways such as the T cell receptor signaling pathway and the B cell receptor signaling pathway. Epididymitis frequently causes male infertility, leading to permanent oligospermia or azoospermia, which is associated with the immune characteristics of the epididymis in up to 40% of patients. Therefore, we believe that Leydig_cells1 potentially represents a cellular subpopulation closely related to spermatogenesis. Next, we selected the top 20 markers of Leydig_cells1, and estimated their expression correlation with histone modification genes; most Leydig_cells1-specific marker genes were positively correlated with histone modification genes (Figure 5B). We performed a PPI network analysis for the Leydig_cells1-specific marker genes (Figure 5C). Expression analysis of histone-modifying genes and Leydig cell marker genes in normal and pathological testicular tissues was conducted using immunofluorescence staining of testicular samples acquired from healthy donors and patients with azoospermia. The results revealed significant upregulation of EZH2 (a key histone methyltransferase) and IL6 (a pro-inflammatory cytokine) in azoospermic testes compared to normal controls. Additionally, HDAC2 showed significant upregulation in azoospermic testes. These findings reflect the strong positive correlation between EZH2 and IL6 expression demonstrated through the transcriptomic co-expression network (Figures 5D–F), suggesting potential crosstalk between epigenetic regulation and inflammatory pathways involved in the pathogenesis of azoospermia. Moreover, we detected HDAC2 upregulation in azoospermic testes compared to that in normal controls (Supplementary Figure S2), which further indicates its importance in azoospermia.

#### Analysis of the signaling pathways and mechanisms of histone modifications that promote disease progression

**FIGURE 6:**
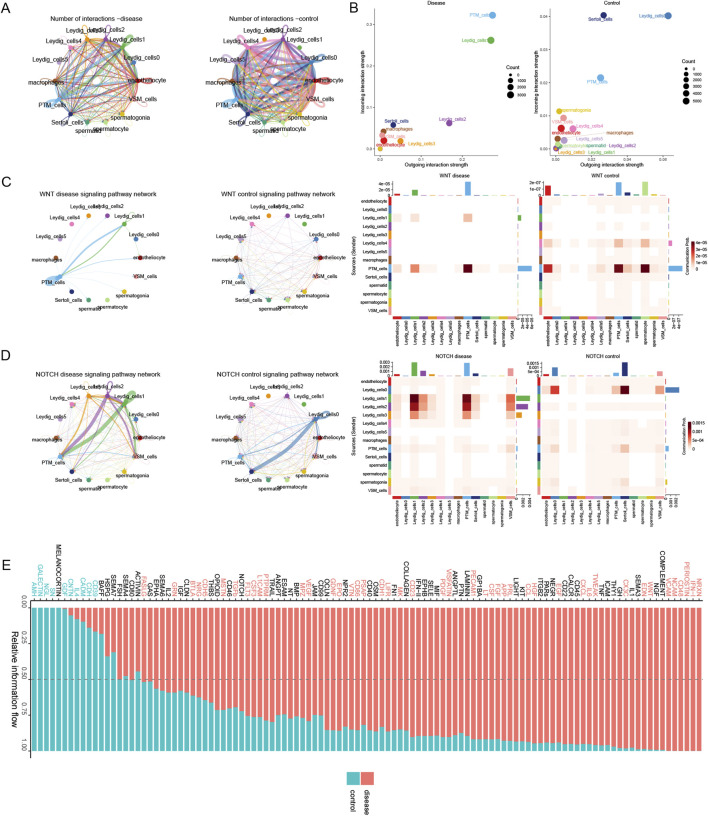
Deciphering the mechanisms underlying histone modification-driven disease progression via cellular communication. **(A)** Overall intercellular communication in the disease and control groups. **(B)** Comparison of the input and output interaction strengths among all cell types in the disease and control groups. **(C)** Variation in the WNT signaling pathway between the disease and control groups. **(D)** Differences in the NOTCH signaling pathway between the disease and control groups. **(E)** The relatively prominent signal communications in the disease and control groups.

We comparatively analyzed cellular communications in the disease and control groups. The interactions among Leydig_cells1 and between Leydig_cells3 and other cells were stronger in the disease group than in the control group. Conversely, Leydig_cells0, Leydig_cells4, and Leydig_cells5 exhibited weaker interactions with other cells, and the functional strength of Sertoli_cells was lower in the disease group than in the control group (Figure 6A). By comparing the input and output interaction strengths of all cell types between the disease and control groups, we demonstrated that the input and output interaction strengths of PTM_cells and Leydig_cells1 cells with testicular interstitial cells were higher in the disease group than in the control group. However, the input and output interaction strengths of Leydig_cells0 and Sertoli_cells were higher in the control group than in the disease group (Figure 6B). Analyses of the histone modification-related signaling pathways revealed that the WNT signaling pathway exhibited a stronger interaction between PTM_cells and Leydig_cells1 in the disease group (Figure 6C). The NOTCH signaling pathway exhibited stronger interactions among PTM_cells, Leydig_cells2, and Leydig_cells1 in the disease group than in their control counterparts. In contrast, interactions between Leydig_cells0 and Sertoli_cells were stronger in the control group than in the disease group (Figure 6D). Finally, we characterized the crucial signaling pathways in both the disease and control groups (Figure 6E).

## Discussion

NOA is one of the most severe forms of male infertility; however, its etiology and mechanisms remain unclear. Azoospermia is frequently associated with varicocele and reproductive tract infections (Jensen and Ko, 2021). These conditions are frequently associated with a decrease in the total sperm count, sperm concentration, and sperm motility, with a profound impact on the patient and their family. Recent global advances in medical technology have provided significant insights into the molecular aspects of male infertility. Currently, testicular biopsy with pathological diagnosis is considered the “gold standard” for assessing spermatogenic function in testes. However, invasive diagnostic procedures can lead to certain complications. Therefore, the search for potential non-invasive molecular biomarkers is considered a major challenge in clinical research.

This study presents a comprehensive analysis of histone modifications in key cellular subpopulations in NOA, based on single-cell RNA sequencing data. Our findings revealed significant heterogeneity in histone modification patterns across various cell types, with significant differences between patients with NOA and healthy controls.

HDAC2, a member of the histone deacetylase family, primarily functions to reduce histone acetylation levels. During spermatogenesis, histone deacetylation is a critical regulatory step for chromatin structural changes, facilitating chromatin remodeling and the tight packaging of sperm DNA. Studies indicate that HDAC2 may be an essential gene in spermatogenesis, potentially regulating gene expression during meiosis, particularly in the prophase (Kui et al., 2019; Ou et al., 2025). For instance, HDAC2 interacts with the chromatin domain protein Cdyl to participate in chromatin remodeling during spermatogenesis. The carboxy-terminal region of Cdyl shares homology with HDAC2 and inhibits its ability to bind coenzyme A (CoA), thereby modulating histone acetylation status and influencing gene expression patterns (Caron et al., 2003). Consequently, upregulation of HDAC2 may contribute to the development of NOA. Our results highlight the role of Leydig cells as a key cellular subpopulation associated with histone modifications in NOA. The enrichment of histone modification-related genes in these cells suggests their potential role in disease pathogenesis. We identified significant upregulation of HDAC2 in Leydig and peritubular myoid cells (PTM cells) in the disease group (Liu et al., 2019). HDAC2, a regulator of histone acetylation, has been implicated in the regulation of gene expression and spermatogenesis. Its dysregulation potentially contributes to spermatogenic defects in NOA. Immunofluorescence staining of the testes revealed that the histone methyltransferase EZH2 and Leydig cell marker gene IL6 or HDAC2 were upregulated in azoospermia. EZH2, a PRC2 enzyme that silences genes via H3K27me3, is highly expressed in the testes and crucially regulates spermatogenesis, stem cell self-renewal, and differentiation (Cao et al., 2002; Lambrot et al., 2012; Zhou et al., 2015; Batool et al., 2019). EZH2 knockout disrupts stem cell maintenance, triggers differentiation/apoptosis in mice, and inhibits tumor growth (Jin et al., 2017). Elevated IL- 6 levels can impair spermatogenesis and fertility through multiple mechanisms: disrupting the blood-testis barrier, inhibiting meiotic DNA synthesis, affecting the permeability of Sertoli cell tight junctions, modulating the secretion of transferrin and inhibin B, reducing sperm motility, and suppressing testosterone secretion by Leydig cells (Potiris et al., 2025; Alves-Silva et al., 2021). In ER-negative cells, EZH2 forms a complex with NF-κB subunits RELA/RELB, directly binds the IL6 promoter, and activates its transcription independently of its methyltransferase activity (Völkel et al., 2015). Within the bone marrow microenvironment, adipocyte-derived IL-6 and TNFα enhance EZH2 expression in myeloma cells (Zhu et al., 2023). Similar mechanisms may operate in the testes. Elevated IL-6 levels are commonly observed in male infertility and induce testicular oxidative stress. These findings indicate a potential crosstalk between epigenetic regulation and spermatogenesis in azoospermia. Furthermore, EZH2 exhibits a nonclassical regulatory role in spermatogonial differentiation and apoptosis in murine spermatogenesis (Jin et al., 2017).

Functional enrichment analysis of DEGs in Leydig cells indicated pathways involved in oxidative phosphorylation, mitochondrial translation, and immune-related functions. This suggests the potential impact of histone modifications on energy metabolism and immune responses in Leydig cells; these processes are crucial for maintaining testicular function and sperm production. The enrichment of PI3K-Akt signaling and apoptotic regulatory pathways in certain Leydig cell subpopulations further supports the hypothesis that histone modifications may be involved in the regulation of cell survival and death, potentially affecting spermatogenesis. PPI network analysis of Leydig cell markers revealed a complex interplay among genes related to spermatogenesis and histone modifications, underscoring the potential of histone modification enzymes for influencing multiple signaling pathways and affecting cellular communication and functions in testicular cells.

Moreover, we compared the cellular communication between the NOA and control groups. The variable interaction strengths between Leydig cells and other cell types, which included the differential activation of WNT and NOTCH signaling pathways, suggest that histone modifications may modulate cell-cell communication and influence disease progression. Specifically, dysregulation of the WNT signaling pathway leads to cytoplasmic accumulation of β-catenin in Leydig cells, inhibiting its nuclear translocation and subsequent target gene expression. This cascade further impairs testosterone synthesis by downregulating StarD7 (Zhang et al., 2018). Studies also indicate that Wnt4 signaling originating from Sertoli cells suppresses the activity of endogenous spermatogonial stem cells (SSCs). This inhibitory effect may trigger germ cell death, consequently disrupting normal spermatogenesis (Liu et al., 2024; Boyer et al., 2012). Furthermore, the Wilms tumor 1 (WT1) transcription factor participates in spermatogenesis regulation via the WNT signaling pathway (Zheng et al., 2014). Models with WT1 deficiency exhibit abnormal accumulation of undifferentiated spermatogonia within the testis and failure of germ cells to complete meiosis (Gao et al., 2006). Mechanistic studies reveal that WT1 loss potentially downregulates NOTCH signaling activity through paracrine mechanisms, thereby perturbing cellular differentiation programs (Wen et al., 2016). Conversely, NOTCH pathway overexpression has been shown to induce premature germ cell differentiation accompanied by progressive apoptosis, ultimately resulting in spermatogenic failure (Garcia et al., 2017). Importantly, WNT and NOTCH signaling exhibit functional crosstalk (e.g., β-catenin/NICD interaction) within Leydig cells and peritubular myoid cells (PMCs). Their synergistic dysregulation amplifies cellular dysfunction (Acar et al., 2021; Xue et al., 2025). The WNT pathway that significantly contributes to stem cell maintenance and differentiation may be particularly correlated with the regulation of spermatogenic cell populations (Li et al., 2024; Gao et al., 2024). The NOTCH pathway is a key regulator of cell fate decisions and has been implicated in the maintenance of spermatogonial stem cells (Moreno Acosta et al., 2023; Parekh et al., 2019).

In conclusion, our study provides novel insights into the role of histone modifications in NOA, suggesting potential target cellular subpopulations and signaling pathways for its effective therapeutic intervention. The identification of specific histone modification patterns in Leydig cells and their correlation with spermatogenesis offers a potential avenue for developing diagnostic biomarkers and therapeutic strategies for male infertility associated with NOA.

Further studies validating the functional significance of these histone modifications *in vitro* and *in vivo* are needed to reveal the potential of epigenetic therapies in male infertility. Additionally, a deeper understanding of the crosstalk between histone modifications and non-coding RNA molecules may reveal novel layers of regulation in spermatogenesis, offering new targets for intervention.

## Data Availability

The data that support the findings of this study are available in GEO datasets (GEO accession number GSE149512).
