# Supplementary figures and images for: Analysis of histone modifications in key cellular subpopulations in the context of azoospermia using spermatogenic single-cell RNA-seq data
Source: Front Bioinform. 2025 Jul 18;5:1626153. doi: 10.3389/fbinf.2025.1626153 (PMC12313672; doi:10.3389/fbinf.2025.1626153)

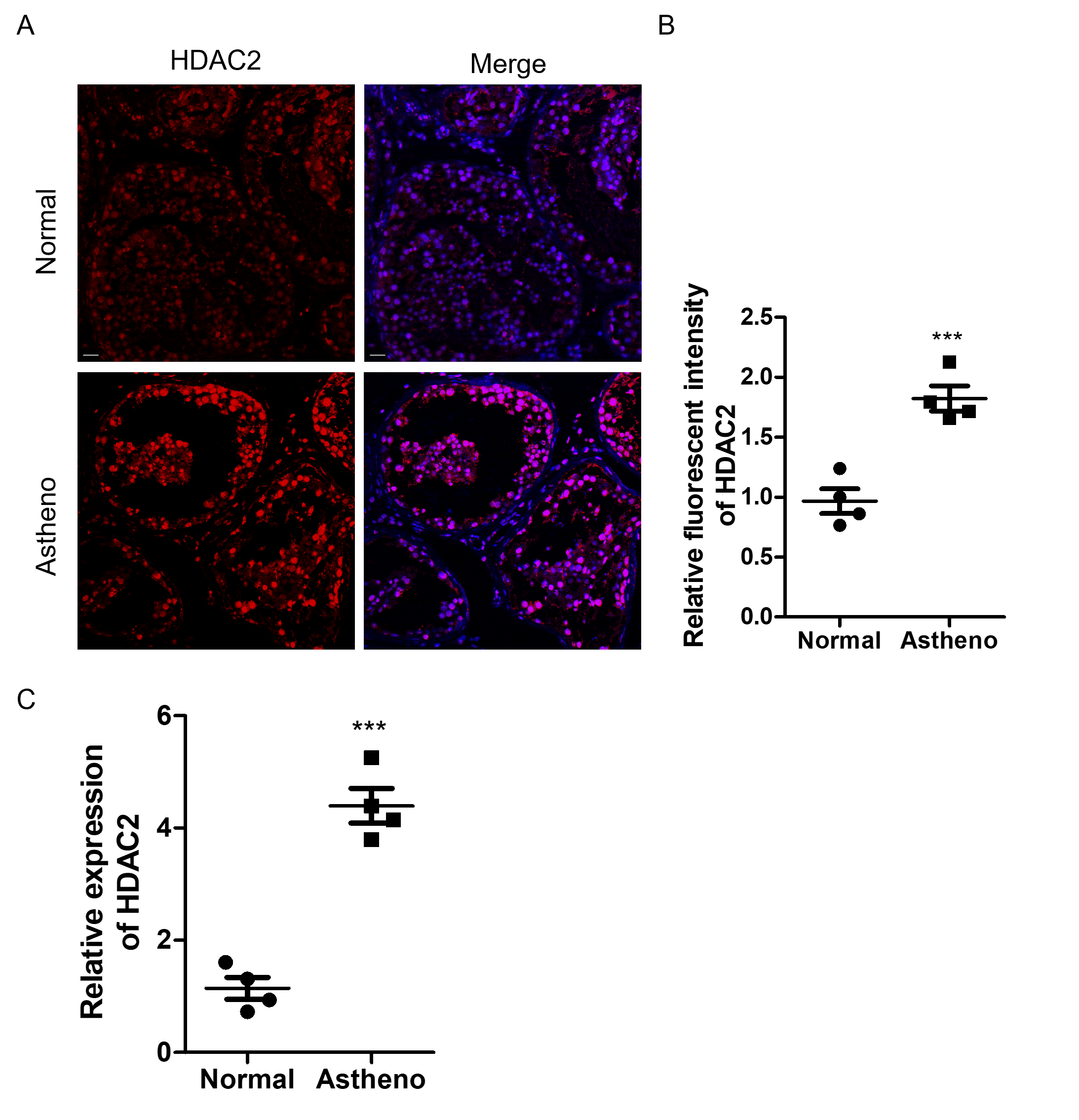

Supplement: Supplementary file 1 [file Image2.tif]

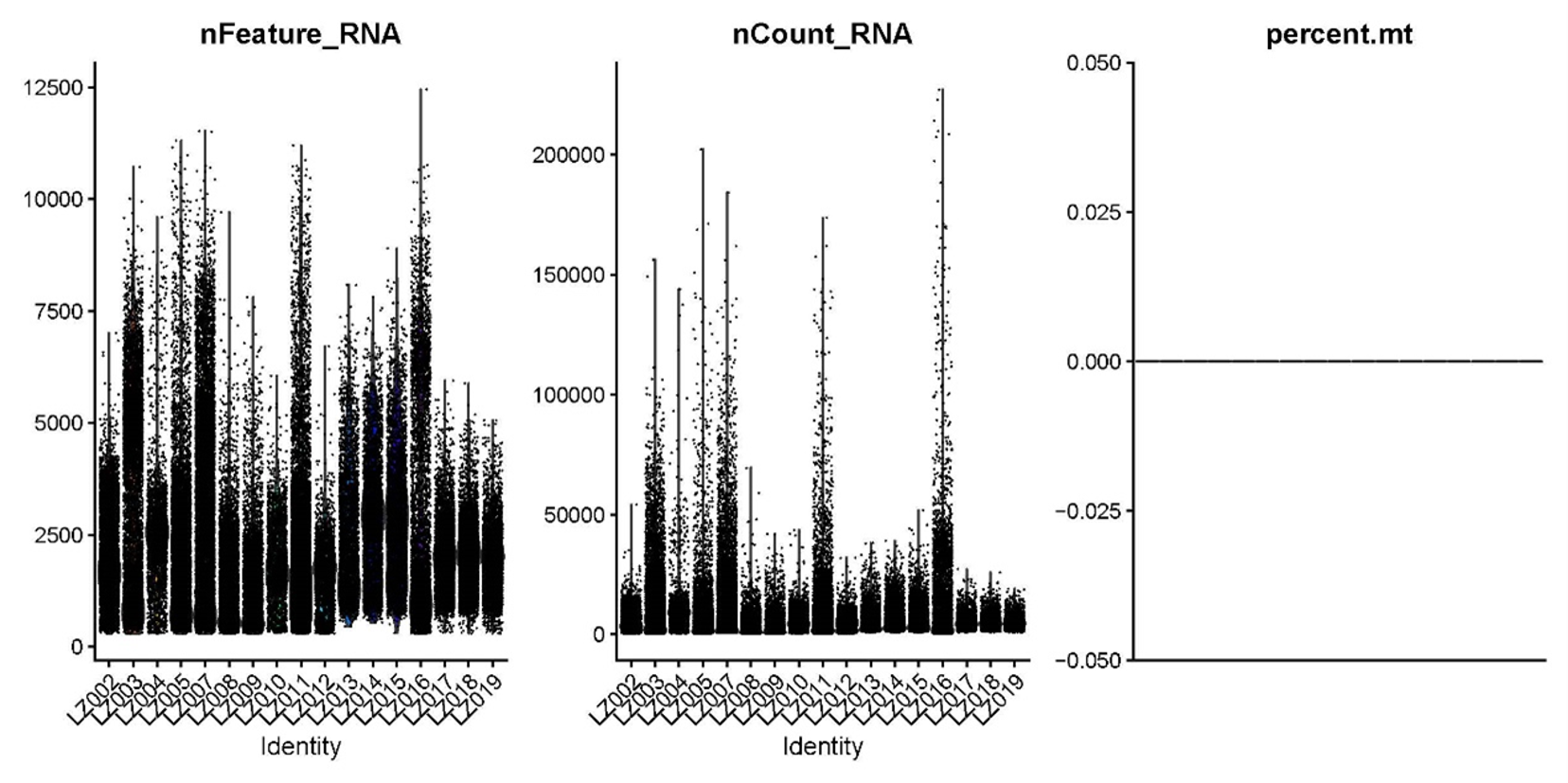

Supplement: Supplementary file 2 [file Image1.tif]
